# Invasive Populations of the Emerald Ash Borer *Agrilus planipennis* Fairmaire, 1888 (Coleoptera: Buprestidae) in Saint Petersburg, Russia: A Hitchhiker?

**DOI:** 10.3390/insects13020191

**Published:** 2022-02-11

**Authors:** Andrey V. Selikhovkin, Dmitry L. Musolin, Boris G. Popovichev, Sergey A. Merkuryev, Mark G. Volkovitsh, Rimvys Vasaitis

**Affiliations:** 1 Department of Forest Protection, Wood Science and Game Management, Saint Petersburg State Forest Technical University, Institutskiy Per. 5, 194021 Saint Petersburg, Russia; a.selikhovkin@mail.ru (A.V.S.); musolin@gmail.com (D.L.M.); b.g.popovichev@yandex.ru (B.G.P.); 2 Pushkov Institute of Terrestrial Magnetism of the Russian Academy of Sciences, Saint Petersburg Filial, Universitetskaya Emb. 5, 199034 Saint Petersburg, Russia; sam_hg@hotmail.com; 3 Institute of Earth Sciences, Saint Petersburg State University, Universitetskaya Emb., 7–9, 199034 Saint Petersburg, Russia; 4 Zoological Institute, Russian Academy of Sciences, Universitetskaya Emb., 1, 199034 Saint Petersburg, Russia; polycest@zin.ru; 5 Department of Forest Mycology and Plant Pathology, Swedish University of Agricultural Sciences, SE-75007 Uppsala, Sweden

**Keywords:** the emerald ash borer, *Fraxinus* spp., forest pests, invasive populations, Northwest Russia, Saint Petersburg, urban pests

## Abstract

**Simple Summary:**

The emerald ash borer (EAB) is an invasive beetle of Asian origin that has killed millions of ash trees in North America and Russia, with a devastating economic and ecological impact. In September 2020, EAB was detected for the first time in Saint Petersburg, Russia, notably killing ash trees. The invasion came from the eastern direction (Moscow) and became a significantly notable event for Saint Petersburg, famous for its historical parks. Moreover, Saint Petersburg is 120–130 km from the eastern EU borders of Estonia and Finland, with railway, motorway, and ferry connections. Currently, EAB is one of the most serious quarantine insect pests in the EU. There is a risk that the eventual EAB invasion could potentially extirpate European populations of ash. Currently, 95% are devastated by the invasive fungal disease ash dieback. Here, we investigated the development of EAB populations in Saint Petersburg, from its initial invasion (estimated year 2015), until 2021. We found that climatic conditions of north Russia do not favor the natural aerial spread of EAB. The two isolated populations were located, respectively, close to a motorway, and the Neva River (used for cargo shipping), implying that the insect spreads by transport vehicles, or “hitchhiking”. This could potentially lead to the eventual invasion of the EU by this serious, tree-killing beetle.

**Abstract:**

The emerald ash borer (EAB), *Agrilus planipennis*, is an invasive beetle of East Asian origin that has killed millions of ash trees (*Fraxinus* spp.) in North America and Russia. In September 2020, EAB was detected in Saint Petersburg, a notable event for the metropolitan city. The aim of the present study was to investigate the occurrence and ecology of EAB in Saint Petersburg. The presence of two distinct enclave populations of EAB was revealed, each of which has, most likely, been established through separate events of “hitchhiking” via transport vehicles. Following the invasion, the further spread of EAB in Saint Petersburg was slow and locally restricted, most likely due to climatic factors. This spread by “hitchhiking” suggests that the possibility of the further long-distance geographic spread of EAB in the Baltic Sea region (the EU) is high, both by ground transport (120–130 km distance from EU borders) and ferries that transport cars across the Baltic Sea. In certain cases, the development of EAB on *Fraxinus excelsior,* based on the stem portion colonized, larval densities, number of galleries, exit holes, viable larvae, and emerged adult beetles, was more successful than in *Fraxinus pennsylvanica* trees. The observed relatively high sensitivity of *F. excelsior* to EAB, therefore, casts doubt on the efficacy and benefits of the currently ongoing selection and breeding projects against ash dieback (ADB) disease, which is caused by the fungus *Hymenoscyphus fraxineus*. Inventory, mapping, and monitoring of surviving *F. excelsior* trees infested by both ADB and EAB are necessary to acquire genetic resources for work on the strategic long-term restoration of *F. excelsior*, tackling the probable invasion of EAB to the EU.

## 1. Introduction

Ash (*Fraxinus* spp.) represents the tree genus that was historically widely used in various kinds of urban greening of Saint Petersburg, where it was commonly planted along streets, alleys, parks, squares, inner gardens, etc., thus becoming one of the principal landscape components of the metropolitan city [1,2]. In the flora of the city and its suburbs, ash is represented by two species, the native European ash *Fraxinus excelsior* L. and the introduced green ash *F. pennsylvanica* Marchall. *Fraxinus pennsylvanica* dominates in the abovementioned urban greening of general/public use, comprising 6.7% of the area, while *F. excelsior* is a typical component of historical parks (such as Peterhof, Gatchina, Tsarskoye Selo, and others) and occasionally grows in mixed forests outside the city [1,2]. The former ash species are encountered only occasionally in those parks, where they are represented by single trees.

The emerald ash borer (EAB), *Agrilus planipennis* Fairmaire, 1888 (Coleoptera: Buprestidae), is a lethal, invasive beetle of East Asian origin that has killed untold millions of ash trees in North America, with a devastating economic and ecological impact [3]. EAB was first recorded in Moscow in 2003 and rapidly produced a massive outbreak in the city, killing most of the ash trees in parks and roadside plantings. Furthermore, the pest started to spread from Moscow in all directions, and by 2020 was recorded in 18 provinces of European Russia, towards the west approaching the border of Belarus, and in the southwest invading eastern Ukraine [4,5]. In September 2020, EAB was detected in Saint Petersburg [5,6,7]. This was a notable event for the city, famous for its architecture and historical parks.

In both European Russia and North America, the formation of EAB invasive enclave populations spatially/geographically separated from the continuous distribution range of EAB is not uncommon [5]. Indeed, one example of such a population is the currently observed invasion of EAB in Saint Petersburg. Previous investigations along the highway from Moscow to Saint Petersburg showed a continuous population stretching westwards from Moscow to Tver [8]. However, no further consistent territorial expansion of the beetle was detected that could be explained by beetles flying over 520 km from Tver to Saint Petersburg [7]. To establish those enclave populations of EAB, long-distance, human-assisted transportation of the beetle by cars and trains was suggested to be necessary [5,7].

Data on the eventual further local spread of invasive populations of EAB in newly invaded enclave geographic areas in Europe are not yet available [5]. Notably, in Saint Petersburg, both EAB and its host tree (ash) coexist at their virtual northern limits, where low winter temperatures and cold and wet summers are common. Moreover, data regarding the consequences of infestation by EAB on *F. excelsior* are scarce and fragmented. Interestingly, in a study under controlled conditions, the frequency with which larvae of EAB developed to later instars in *F. excelsior* was much lower than in the highly susceptible American black ash *Fraxinus nigra* Marchall [9]. Furthermore, during the extensive survey of EAB in western Russia and eastern Ukraine, an overwhelming majority of the infestations was found on the highly susceptible American *F. pennsylvanica*, and all observed cases of infestation of the native species *F. excelsior* occurred in artificial plantings [4].

Consequently, more detailed studies are needed regarding the relative susceptibility of *F. excelsior* to the pest compared to *F. pennsylvanica*. Although there are certain indications that *F. excelsior* might be more resistant to EAB in this respect, e.g., along roadsides and city plantings [10,11], a recent study conducted in the Moscow Province provided evidence that EAB can also cause massive outbreaks and significant damage in forest stands [5]. The aims of the present study were to investigate the population of EAB in the city of Saint Petersburg, Russia, and to evaluate whether temporal trends in temperature might have contributed to the insect’s establishment success.

## 2. Materials and Methods

### 2.1. Survey

**Figure 1:**
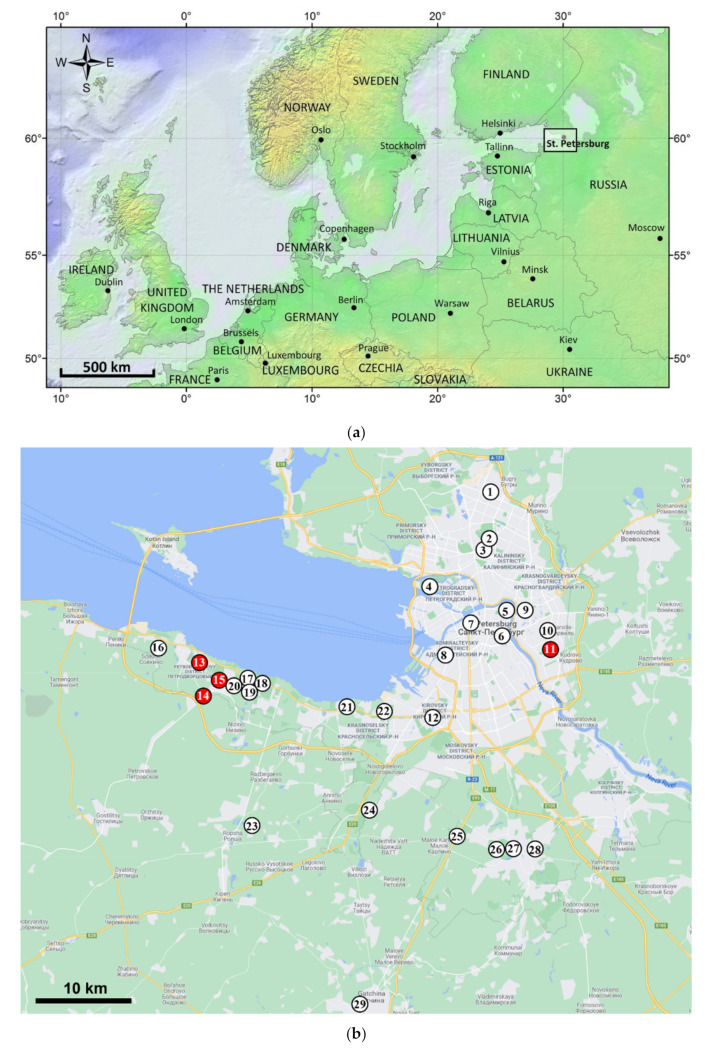
Location of the survey. (**a**) A schematic map of northern and eastern Europe with a rectangle outlining the survey area in Saint Petersburg; (**b**) Localities in the territory of Saint Petersburg at which the survey for the emerald ash borer has been conducted in 2020 and 2021. Location numbers are the same as in Table 1. Red circles indicate locations at which the beetle was detected, while white circles represent locations at which it was not detected. Location # 11 is situated in the Nevsky District, and locations # 13–15 are in the Petrodvortsovy District. Locations # 17–21 are situated in the territory of the Peterhof State Museum Reserve. A basic map used to create Figure 1b is based on an image from © Google, 2021.

In order to check for attacks of EAB on ash trees in the area of Saint Petersburg (Russia) (Figure 1a), from September 2020 to July 2021, we surveyed urban plantings and city parks (Figure 1b; localities # 1–15, 22, and 24–28), and the Peterhof (Petrodvorets) and the Gatchina State Museum Reserves (Figure 1b; localities # 16–21, 23, and 29). ArcGIS Desktop: Release 9.3 was used to prepare a schematic map (Figure 1a).

Sites for the investigation were assigned following the suggestions and guidance by the staff of the Department of Gardening and Park Management, Saint Petersburg City Administration, based on maps of the managed urban greening (city parks, squares, alleys, and streets) in which ash plantings comprised compact groups. This allowed our investigation to cover ash populations of Saint Petersburg to the most comprehensive extent. In each of the investigated plantings, ash trees were investigated in a simple systematic manner: after inspection of the first tree (located at planting edge), the next nearest tree was inspected, and so on, while in certain plantings, each ash tree was inspected. Solitary, in most cases rotten, trees, posing a hazard to public and property (e.g., to by-passers, cars, houses, electricity lines, etc.), were assigned for felling by the Department staff. Single trees growing nearby, were also investigated. Throughout the paper, these two types of sites are referred to as “localities” (Figure 1b; Table 1).

Investigations in the Peterhof and Gatchina State Museum Reserves were conducted with the permission of their respective administrations. Here, *F. excelsior* was historically planted in large, spatially separated, compact groups that, in our case, represented localities of the study (Figure 1). In those, the survey design was largely similar to that described above for city plantings, only comprising larger numbers of inspected trees (e.g., 275, …, 1500) (Table 1). Smaller groups of ash growing in the vicinity were also inspected.

The study included a total of 29 localities distributed throughout the city of Saint Peterburg and its suburbs (Figure 1b). Studied localities represented different types of ash (*Fraxinus* spp.) plantings in the urban environment, such as city parks, squares, alleys, streets, roadsides, and two historical parks—Peterhof (Petrodvorets) and Gatchina. In total, 185 *F. excelsior* and 222 *F. pennsylvanica* trees were visited and inspected. Trees in the Peterhof Park were almost exclusively (2409) *Fraxinus excelsior*, with only three *F. pennsylvanica*, and in the Gatchina Park, all 550 ash trees were *F. excelsior*. The study included 3144 *F. excelsior* trees and 225 *F. pennsylvanica*, comprising 3369 trees in total (Table 1).

**Figure 2:**
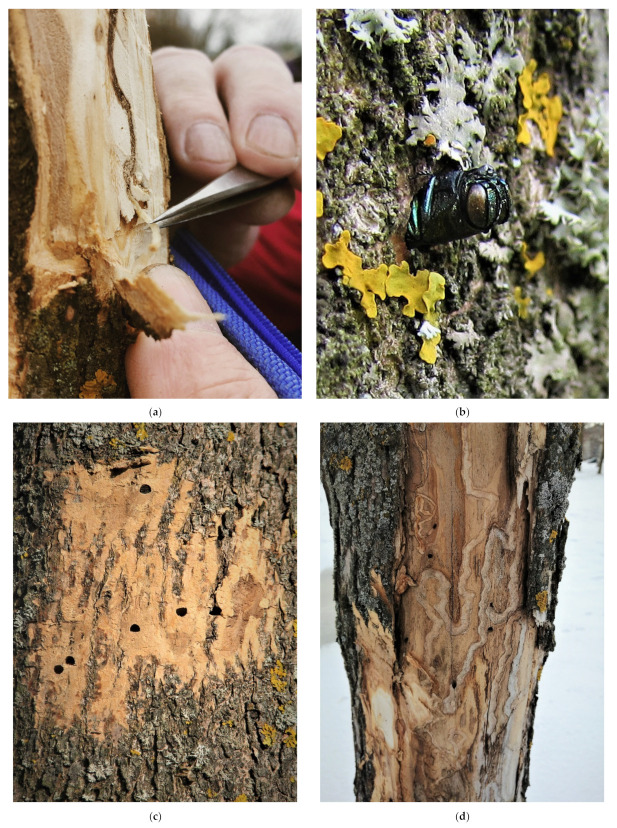
The emerald ash borer, *Agrilus planipennis* on ash trees in Saint Petersburg. (**a**) A larva being extracted from under the bark of *Fraxinus pennsylvanica*; (**b**) an adult emerging through an exit hole; (**c**) exit holes in the bark of *F. pennsylvanica*; (**d**) old galleries of the beetle on the dry-sided stem of *F. pennsylvanica*. Photo by D.L. Musolin (**a**), M.G. Volkovitsh (**b**,**c**) (Location # 13.1), and A.V. Selikhovkin (**d**) (Location # 14).

During the investigations, the lower part of the tree stems was visually inspected for incidence of bark loosening and cracks and the presence of characteristic exit holes of adult beetles and galleries of their larvae (Figure 2). The upper part of a stem and thick branches were inspected using Zeiss TERRA ED 8 × 32 binoculars (Oberkochen, Germany). Each tree where the attack of EAB was detected was felled and examined in detail. We followed routine practices of the Saint Petersburg City Administration for the removal of ash trees with various types of damage that represent a potential threat to the public and property. The number of trees examined, types of plantations, and GPS coordinates of localities are given in Table 1.

### 2.2. Dating Infestations of EAB

Dating infestations of EAB was accomplished for the following three categories of trees:

(1) *Fraxinus pennsylvanica* in the urban greenings of the Petrodvortsovy District, 20–62 cm diameter at breast height (DBH), a total of 78 trees, planting dates unknown (sublocality # 13.1 and locality # 14);

(2) *Fraxinus pennsylvanica* in Nevsky District, 16–24 cm DBH, a total of 19 trees planted in 1991 (sublocality # 11.1);

(3) *Fraxinus excelsior* in Nevsky District, 11–12 cm DBH, a total of 35 trees planted in 2018 (sublocality # 11.2) (Figure 1b).

For the trees from the first two categories, an attempt was made to distinguish between “old” and “new” attacks of EAB, and to estimate the timing of those, while for trees from the third category, only the timing of recent attacks could be estimated. The basis for the abovementioned estimates was the visual examination of larval galleries, exit holes, and the eventual presence of their larvae. The following criteria were applied. “Old”: the presence of exit holes, dry-sided part of a stem, empty galleries, empty pupal chambers, dry and dark cambium. “New”: the absence of exit holes, the presence of larvae in galleries and/or pupae in pupal chambers, light-colored moist cambium.

### 2.3. Examination of Sample Trees

During January–April 2021, seven trees infested by EAB were subjected to individual, detailed investigations as sample trees. These included four *F. pennsylvanica* trees 16–24 cm DBH (category 2; sublocality # 11.1), and three *F. excelsior* trees 11–12 cm DBH (category 3; sublocality # 11.2). They were felled, and for each of them, the proportion of the stem colonized by EAB was estimated based on the presence of exit holes and galleries. Trees in category 2 were subsequently cut into stem sections 0.5 m in length, and trees in category 3 into the sections 1 m in length. For each section, bark surface area was estimated, as well as the numbers of exit holes of EAB, individual galleries, and live larvae. For trees in category 2, based on the external condition and length and width of the galleries, periods of infestation of different parts of stems were estimated.

### 2.4. Analysis of Temperatures

The temporal trends in temperatures in the Saint Petersburg urban area were investigated using data from the World Data Centre of the All-Russian Research Institute of Hydro-Meteorological Information (RIHMI–WDC). Regression analysis was performed on absolute minimum temperatures and the sum of effective temperatures (i.e., temperatures above the lower developmental threshold of 10 °C) recorded during 41 years (=40 winter seasons: 1980–2020). Deterministic linear models were fitted using the least-squares method.

### 2.5. Statistical Analyses

The nonparametric Mann–Whitney *U* test was used to examine differences in the larval density and successful development of EAB (the percentage of emerged adult beetles and viable larvae out of all larvae that started to construct galleries) between infested *F. excelsior* and *F. pennsylvanica* trees. To run this analysis, the STATISTICA program, Ver. 7.0 (TIBCO Software Inc., Palo Alto, CA, USA), was used.

## 3. Results

### 3.1. The Occurrence of EAB in Saint Petersburg

EAB was detected in two distinct areas, one comprising locality # 11 in the Nevsky District and another comprising the cluster of localities # 13–15 in the Petrodvortsovy District (Figure 1). As is evident from Figure 1, these two districts are geographically separated from each other by approximately 40 km, passing in a straightforward manner partly through the Finnish Gulf and through the central part of Saint Petersburg, which includes 13 localities (# 5–10, 12, and 17–22) where EAB was not detected. Furthermore, EAB was also not detected at any of the other 12 urban localities investigated, nor in the Peterhof and Gatchina parks (Figure 1; Table 1).

The locality where the infestation was recorded in the Nevsky District was comprised of two sublocalities—a city park and an alley. *Fraxinus pennsylvanica* was the only ash species represented in the city park, and among its 28 trees, 19 (67.9%) were infested by EAB. The neighboring alley was composed exclusively of 35 *F. excelsior* trees, 8 of which (22.9%) were infested by EAB. The localities where the infestation was recorded in the urban Petrodvortsovy District represented plantings along roads and streets and a square (an inner garden). Here, out of a total of 110 *F. pennsylvanica* trees examined, 83 (75.5%) were infested by EAB, while one of the three *F. excelsior* trees was infested (Table 1). Examples of records of living larvae, emerging adult beetles, exit holes, and old galleries of EAB observed on stems are presented in Figure 2.

In sublocality # 11.1 belonging to the Nevsky District, *F. pennsylvanica* trees growing in the park were planted in 1991, while the alley in sublocality # 11.2 consisted exclusively of younger *F. excelsior* trees planted in 2018. During the investigation time, *F. pennsylvanica* in sublocality # 11.1 had reached a stem DBH of 16–24 cm. Among those trees, parts of the stems infested by EAB were dry-sided and had relatively old galleries of EAB (Figure 2d). On the other hand, on stems of the same *F. pennsylvanica* trees, a rather clear distinction was noted between old galleries observed in the lower parts of the stem and the fresh ones located higher up. Only fresh galleries of a similar age were observed at the neighboring sublocality # 11.2 on young *F. excelsior*. Furthermore, a rather similar situation was observed in the Petrodvortsovy District (sublocality # 13.2 and locality # 14), where older EAB-infested trees with a DBH of 20–62 cm had dry-sided stems with old galleries, but newly planted trees with a DBH of 8–12 cm were recently attacked by EAB.

### 3.2. Ecology of a Local Population of EAB in the Nevsky District

No significant difference in larval density (Mann–Whitney *U* test; U = 18.5; *p* > 0.05) and successful larval development of EAB (the percentage of emerged adult beetles and viable larvae out of all larvae that started to construct galleries) (Mann–Whitney *U* test; U = 11.0; *p* > 0.05) was found between the two examined *Fraxinus* species (Table 2). Moreover, Table 2 demonstrate the approximate extent of colonization of four *F. pennsylvanica* sample stems by EAB in sublocality # 11.1, which differs between the four investigated trees.

During the first infestation wave in each stem from sublocality # 11.1, EAB colonized approximately similar stem portions: up to 4 m height in three stems (nos. 1–3) and up to 2 m in stem no. 4. Yet, the situation changed following the second infestation wave, as in this instance, newly colonized parts of stems differed sharply in height: in tree no. 1 zero colonization, in no. 2–1 m, in no. 3–2 m, while in tree no. 4–7 m. Thus, after the two waves of infestation, colonized portions in those stems comprised in total 4, 5, 6, and 9 m, respectively (Table 2).

Notably, in three neighboring *F. excelsior* trees (nos. 5–7) (sublocality # 11.2), subject only to the second wave of infestation by EAB, in a vertical direction 3, 5, and 6 m of the stems were colonized, resulting in a comparable extent of colonization to that recorded in *F. pennsylvanica* trees after two infestation waves (Table 2). It must be emphasized, however, that in the present study, empirical data for accomplished comparisons and statements drawn is extremely small.

The same applied regarding absolute numbers of larval densities, larval galleries, exit holes, viable larvae, and emerged adult beetles of EAB, which, in each comparison, were higher in *F. excelsior* (subjected only to the second infestation wave) than in *F. pennsylvanica* (subjected to both infestation waves) (Table 2).

## 4. Discussion

The results show that initial outbreaks of EAB both in the Nevsky and Petrodvortsovy Districts occurred in 2015 and continued up to 2018, followed by a new outbreak of EAB that in both areas took place in 2019 on the newly planted ash, one year after outplanting. Moreover, the current study provides evidence for the existence of two distinct areas of the outbreak of EAB in Saint Petersburg. The exact modes of entry of EAB to each of those are hard to define, yet the possibility cannot be excluded that they are of different origins. Notably, in the Peterhof’s (Petrodvortsovy District) locality # 14 infested *F. pennsylvanica* comprised planted rows of trees in the vicinity of a busy highway, while the Nevsky locality # 11 is situated in the opposite part of the city close to the River Neva, which is commonly used as a water transport (shipment) corridor for wood to the Gulf of Finland. The introduction of EAB with plants for planting seems unlikely. In both areas, initial infestations are old and estimated to have started in 2015, as hypothesized previously [12]. Attacked trees are also old and large, and younger *F. excelsior* treess, e.g., in Nevsky District, were outplanted years after the initial outbreak.

Apart from being situated close to two distant and distinct major transport corridors, the highway and the river, the EAB-invaded localities in Saint Petersburg are geographically separated from each other by approximately 40 km that includes both the Finnish Gulf and the city center, where the ash, in the meantime, has remained infestation-free (Figure 1b). Consequently, the possibility that EAB spreads between the two localities by flying should be excluded. Therefore, this makes it rather likely that each of the detected outbreaks was established by separate events of “hitchhiking”. On the other hand, the possibility cannot be excluded that the invasion of Saint Petersburg by EAB did indeed originate from a single introduction event and that further local spread of the beetle within the city was accomplished by car transport. It was reported that EAB can easily be transported by cars, hidden behind flanges of the car body [13]. However, neither of those two hypotheses can be supported without DNA analyses of EAB samples from different survey localities.

Nevertheless, it is still possible that further long-distance geographic spread of EAB in the Baltic Sea region may occur, not only by ground transport (outbreaks located at a 120–130 km distance, and Saint Petersburg is well connected by highways and railroads to Russia’s border with Estonia and Finland) but also by ferries transporting cars and wood, especially bearing in mind that the passenger ferry transport infrastructure across the Baltic Sea is well-developed.

However, following the invasion, the further spread of EAB in Saint Petersburg was slow and locally restricted. One speculative explanation for this could be the low connectivity of *Fraxinus* plantings and the highly urbanized environment of the city. Moreover, our study has demonstrated that EAB in Saint Peterburg has a two-year generation. Another indication of the low suitability of the Saint Petersburg area for the development of populations of EAB is the fact that none of the *F. pennsylvanica* trees attacked during the first infestation wave in 2015–2018 has been killed, thus surviving to the second infestation wave of 2019–2020. The most likely explanation for the restricted EAB spread is climatic factors, in particular low winter temperatures [5,14].

In Saint Petersburg, despite the generally continuous trend of climate warming, temperatures critically low for the development of EAB were recorded in the previous century (e.g., in 1987), but also during the 2000s, the absolute annual minimum decreased to almost −30 °C on severa occasions, while in 2016 the absolute minimum temperature decreased to nearly −25 °C (Supplementary Figure S1). In 2017, the sum of effective temperatures (calculated above 10 °C), at which the development of EAB presumably occurs, also sharply decreased (Supplementary Figure S2).

Absolute annual minimum temperatures below −30 °C are seemingly critical for the survival of populations of EAB, and the isotherm of an absolute minimum of −34 °C is apparently the climatic boundary for the distribution area of EAB [5,14]. Data acquired during the present study indicate that EAB probably actively explored its food resources of ash during the initial stages of the outbreak in Saint Petersburg, but following low temperatures of 2016–2017 (Supplementary Figures S1 and S2), the expansion slowed down. In this case, the thick bark, under which larvae of EAB develop, could serve as an extra barrier of protection against low temperatures. In fact, the lower part of the stems, encased by the thicker bark, is where the development of larvae of EAB has been successfully completed (Table 2).

It is possible that EAB could exhibit enhanced establishment and spread upon arrival in Helsinki, Tallinn, or Stockholm, and that, although they are seaports situated at similar northern latitudes, they have more maritime, thus milder climates [15]. On the other hand, it is hard to extrapolate and interpret our results on a wider geographical scale since the climate is more suitable for the pest in more southerly areas. In the literature, there are numerous examples demonstrating temperature dependencies of invasions of beetles that are pests of forest trees, in particular bark beetles of the genera *Dendroctonus* Erichson, 1836 and *Ips* De Geer, 1775, both in Europe and North America [16].

An important message generated by this work is that, although the data are scarce and fragmented, *F. excelsior* might be even more prone to attacks of EAB than *F. pennsylvanica*, thus contradicting some previous observations [4,17]. Notably, Table 2 demonstrate that larval densities, numbers of larval galleries, exit holes, viable larvae, and emerged adult beetles were slightly, though not statistically significantly, higher in *F. excelsior* following a single wave of infestation than in *F. pennsylvanica* after two waves. The longitudinal extent of stem colonization in both species was similar. As relatively large trees of *F. excelsior* have been planted relatively recently prior to the attacks by EAB, such predisposition towards the pest could be probably explained by post-planting stress. The current study presents the first available numerical/quantitative data documenting attacks by EAB to *F. excelsior*. It also demonstrates that in certain cases following the attack (e.g., due to tree exposure to the sun), the development of EAB in *F. excelsior* might be more successful than that observed in *F. pennsylvanica*.

Since the mid-2000s, severe ash dieback (ADB) was observed in most European countries. The causal agent of ADB is the invasive, alien fungus *Hymenoscyphus fraxineus,* Queloz et al. The disease results in the large scale mortality of *Fraxinus* spp., threatening the existence of this tree genus on a continental scale [18]. Yet, there is evidence that a proportion of *F. excelsior* individuals across European populations exhibits certain tolerance/resistance to ADB, providing the basis for its future selection, breeding, and propagation [19,20]. However, the proportion showing high resistance to ADB is low, estimated to be between 1 and 5% of individuals [10,21,22]. Consequently, ex situ conservation and resistance breeding programs were initiated in many European countries, providing a promising perspective for long-term mitigation of the damaging effects of ADB [18]. However, the large scale infestation of EAB combined with infection by ADB is expected to be more lethal than either of them alone [21]. This raises the question of how effective and beneficial the current selection and breeding programs against ADB will be and to what extent they will contribute to the long-term restoration of native European ash populations, tackling the possible invasion of EAB to the EU.

As EAB continues to spread to the west, it will increasingly encounter trees affected by ADB, and the implications of the interaction will need to be accounted for in developing surveillance and response strategies to the beetle [23]. In this respect, the current study has certain, although so far limited, practical implications, as only ash trees that were subjected to infestations both by ADB and EAB were investigated. *F. excelsior* trees planted in sublocality # 11.2 have been previously subjected to ADB infection pressure (airborne spores) for at least a decade (as all other ash species around the Baltic Sea region) and apparently were free from ADB symptoms after subsequent replanting in an urban alley. Therefore, those 27 *F. excelsior* trees that survived or were not susceptible to attacks by EAB (Table 2; location # 11.2) constitute a source of material for further monitoring and eventual propagation, for which the current work represents a starting point. Similar investigations should be initiated on a large scale in areas invaded by EAB in Russia and eastern Ukraine, comprising a unique genetic resource (e.g., seed banks) for the whole of Europe.

## 5. Conclusions

1. The current work has revealed the presence of two distinct enclave populations of the emerald ash borer (*Agrilus planipennis*) in Saint Petersburg, established either by a single or two separate events of long-distance “hitchhiking” via transport vehicles.

2. Initial outbreaks of EAB, both in the Nevsky and Petrodvortsovy Districts, occurred in 2015 and continued up to 2018, followed by a new outbreak of EAB in both areas that took place in 2019 on the newly planted ash.

3. Following the invasion further spread of EAB was slow and locally restricted, likely due to unsuitable climatic conditions, characterized by cool and wet summers and freezing winter temperatures.

4. EAB exhibited a slightly more successful development in *Fraxinus excelsior* than in *F. pennsylvanica*. Larval densities, numbers of larval galleries, exit holes, viable larvae, and emerged adult beetles were slightly higher in *F. excelsior* following a single wave of infestation than in *F. pennsylvanica* after two waves. The longitudinal extent of stem colonization in both species was similar.

5. The efficacy and expected benefits of currently ongoing European ash selection and breeding projects against ash dieback are under question.

6. Inventory, mapping, and monitoring of surviving ash trees infested both by ADB and EAB are necessary to acquire genetic resources for work on the strategic, long-term restoration of *F. excelsior*, tackling a possible invasion of EAB to the EU.

## Figures and Tables

**Table 1:** Characteristics of localities (numbers are the same as in Figure 1) in the territory of Saint Petersburg where eventual attacks of the emerald ash borer (EAB) (*Agrilus plannipenis*) on *Fraxinus excelsior* and *F. pennsylvanica* trees were inspected. **Localities where the beetle was detected (no. 11, 13, 14, and 15) are printed in bold**.

No.	GPS Coordinates of the Locality	Ash Species ^a^	Plantation Category	No. of Investigated Ash Trees		
				Visually from the Ground	Of Those, Felled and Examined in Detail	Attacked by EAB
**PLANTINGS IN URBAN ENVIRONMENT**						
3	59°59′34″ N, 30°20′33″ E	*F. ex.*	city park	20	0	0
4	59°58′16″ N, 30°14′48″ E	*F. ex.*	city park	6	2	0
5	59°56′41″ N, 30°22′49″ E	*F. ex.*	square	1	1	0
6	59°55′51″ N, 30°22′36″ E	*F. ex.*	square	5	1	0
7	59°54′49″ N, 30°17′17″ E	*F. ex.*	square	4	1	0
8	59°54′13″ N, 30°15′39″ E	*F. ex.*	city park	15	0	0
10	59°51′59″ N, 30°21′50″ E	*F. ex.*	city park	6	1	0
22	59°50′48″ N, 30°08′54″ E	*F. ex.*	city park	15	0	0
26	59°43′23″ N, 30°23′41″ E	*F. ex.*	city park	20	0	0
27	59°43′04″ N, 30°22′11″ E	*F. ex.*	city park	30	4	0 ^b^
28	59°43′10″ N, 30°25′26″ E	*F. ex.*	alley	25	0	0
		Total *F. ex.*		147	10	0
1	60°03′32″ N, 30°20′59″ E	*F. p.*	street	10	0	0
2	60°00′00″ N, 30°21′01″ E	*F. p.*	street	3	0	0
9.1 ^c^	59°55′35″ N, 30°24′26″ E	*F. p.*	square	1	1	0
9.2	59°55′58″ N, 30°24′38″ E	*F. p.*	city park	16	1	0
12	59°50′47″ N, 30°15′32″ E	*F. p.*	street	4	1	0
24	59°43′56″ N, 30°05′32″ E	*F. p.*	city park	10	3	0
25	59°43′50″ N, 30°17′35″ E	*F. p.*	roadside	40	3	0
		Total *F. p.*		84	9	0
**EAB invasion, Nevsky District**						
**11.1 ^c^**	**59°54′31″ N, 30°27′43″ E**	***F. p.***	**city park**	**28**	**19**	**19**
**11.2**	**59°54′38″ N, 30°27′59″ E**	***F. ex*** **.**	**alley**	**35**	**8**	**8**
**EAB invasion, Petrodvortsovy District**						
**13.1 ^c^**	**59°54′08″ N, 29°49′02″ E**	***F. p*** **.**	**street**	**63**	**63**	**63**
**13.2**	**59°54′08″ N, 29°49′02″ E**	***F. ex.***	**street**	**3**	**1**	**1**
**14**	**59°51′49″ N, 29°48′38″ E**	***F. p.***	**roadside**	**15**	**15**	**15**
**15.1 ^c^**	**59°53′01″ N, 29°52′00″ E**	***F. p.***	**square**	**20**	**0**	**4**
**15.2**	**59°53′36″ N, 29°51′54″ E**	***F. p.***	**roadside**	**12**	**0**	**1**
		**Total *F. p.* + *F. ex.***		**110 + 3**	**78 + 1**	**83 + 1**
**PETERHOF STATE MUSEUM RESERVE**						
16	59°54′47″ N, 29°44′36″ E	*F. ex.*	park	600	0	0
17	59°53′12″ N, 29°54′30″ E	*F. ex.*	park	1500	4	0 ^b^
18	59°52′52″ N, 29°56′22″ E	*F. ex.*	park	275	0	0
19	59°52′37″ N, 29°54′29″ E	*F. ex.* + *F. p.*	park	7 + 2	1 + 0	0
20	59°53′33″ N, 29°51′59″ E	*F. ex.*	park	25	0	0
21	59°51′13″ N, 30°02′46″ E	*F. ex.* + *F. p.*	park	2 + 1	0	0
		Total *F. ex*. + *F. p.*		2409 + 3	5	0
**GATCHINA STATE MUSEUM RESERVE**						
23	59°43′24″ N, 29°51′31″ E	*F. ex.*	park	300	12	0 ^b^
29	59°33′47″ N, 30°06′52″ E	*F. ex.*	park	250	35	0 ^b^
		Total *F. ex.*		550	47	0
**THROUGHOUT THE STUDY**						
		*F. ex.*, no. (%)		3144	71	9 (0.3)
		*F. p.*, no. (%)		225	106	102 (45.3)
		ALL, no. (%)		3369	177	111 (3.3)

^a^—ash species: *F. p*.—*Fraxinus pennsylvanica*; *F. ex*.—*F. excelsior*. ^b^—galleries of *Hylesinus crenatus* and *Hylesinus fraxini* (Coleoptera: Curculionidae). ^c^—localities # 9, 11, 13, and 15 are divided into two, as each included two different categories of plantations.

**Table 2:** Infestations of the emerald ash borer (*Agrilus planipennis*) on individual trees of *Fraxinus pennsylvanica* (planted in 1991; sublocality # 11.1) and *F. excelsior* (planted in 2018; sublocality # 11.2). The sublocalities as shown in Figure 1 and Table 1.

Tree No./Years Of Infestation	Ash Species ^a^	DBH ^b^ (cm)	Height (m)	Colonized Stem Height, Min–Max (m)	Larval Density (no./1 dm^2^ of Bark) ^c^	Number per Tree			Percent	
						Larval Galleries	Exit Holes	Viable Larvae	Emerged Adult Beetles	Emerged Adult Beetles + Viable Larvae ^d^
1/2015–2018	*F. p*.	16	8.5	0–4	0.34	58	26	0	44.8	44.8
1/2019–2020				+ 0 ^e^	0	0	0	0	0	0
2/2015–2018	*F. p*.	16	10.2	0–4	0.53	92	44	0	47.8	47.8
2/2019–2020				+ 1 ^e^	0.29	50	10	12	0	44.0
3/2015–2018	*F. p*.	20	12.4	0–4	0.25	64	48	0	75.0	75.0
3/2019–2020				+ 2 ^e^	0.04	10	0	4	0	40.0
4/2015–2018	*F. p*.	21	9.3	0–2	0.14	30	12	0	40.0	40.0
4/2019–2020				+ 7 ^e^	0.04	32	22	8	0	93.8
5/2019–2020	*F. ex*.	11	7.2	0–6	0.29	43	8	26	0	79.1
6/2019–2020	*F. ex*.	12	6.4	0–5	0.71	156	37	75	0	71.8
7/2019–2020	*F. ex*.	11	5.5	1–3	0.77	60	4	3	0	11.7

^a^—ash species: *F. p*.—*Fraxinus pennsylvanica*; *F. ex*.—*F. excelsior*. ^b^—diameter at breast height. ^c^—no statistical difference in larval density between *F. pennsylvanica* and *F. excelsior* (Mann–Whitney *U* test; U = 18.5; *p* > 0.05). ^d^—no statistical difference in successful development (the percentage of emerged beetles and viable larvae out of all larvae that started construction of galleries) between *F. pennsylvanica* and *F. excelsior* (Mann–Whitney *U* test; U = 11.0; *p* > 0.05). ^e^—stem portion (length) newly colonized following the 2nd infestation period.

## Data Availability

Data are available upon email request to the corresponding author.

## Supplementary Materials

The following supporting information can be downloaded at: https://www.mdpi.com/article/10.3390/insects13020191/s1, Figure S1: Absolute minimal temperatures in Saint Petersburg during 1980–2020; Figure S2: Dynamics of the sum of effective temperatures in Saint Petersburg during growing seasons (May–September) in 1980–2020.
