# Supplementary material for: Invasive Populations of the Emerald Ash Borer Agrilus planipennis Fairmaire, 1888 (Coleoptera: Buprestidae) in Saint Petersburg, Russia: A Hitchhiker?
Source: Insects. 2022 Feb 11;13(2):191. doi: 10.3390/insects13020191 (PMC8880411; doi:10.3390/insects13020191)
Supplement: Supplementary file 1 [file insects-13-00191-s001.zip › insects-1475566-supplementary.pdf]

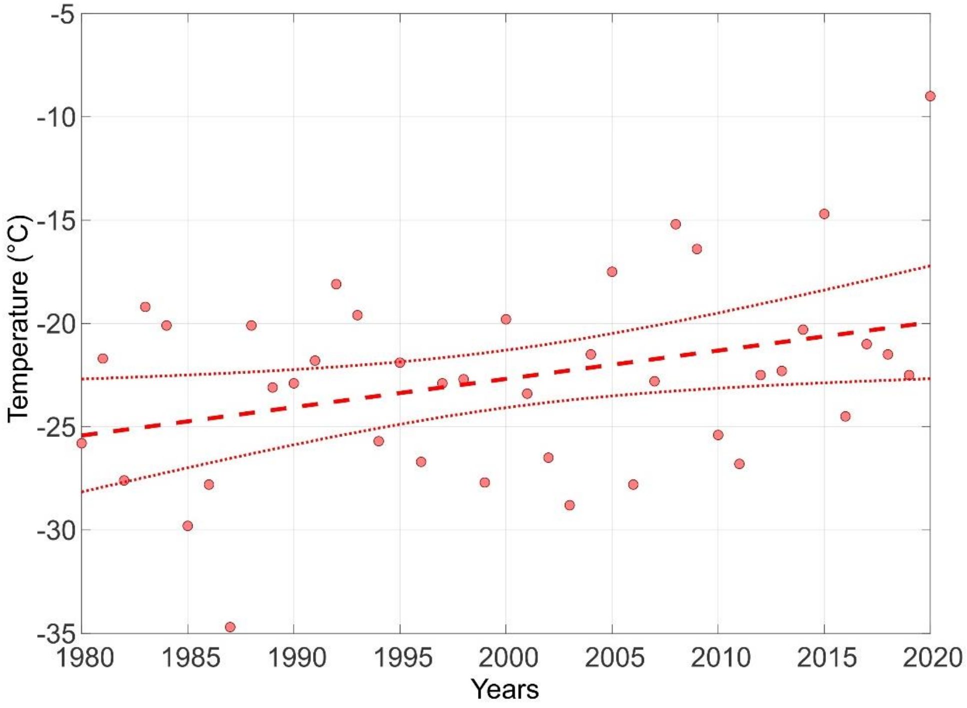

Figure S1. Absolute minimal temperatures in Saint Petersburg during 1980–2020. Pink circles, measured values; dashed line, the line of regression (based on the least squares; y=0.14x–25.6, where y – temperature (°C), x – year; P=0.0235); dotted lines, confidence interval (95% significance level). Correlation coefficient Rcoef=0.35. Data are taken from [15].

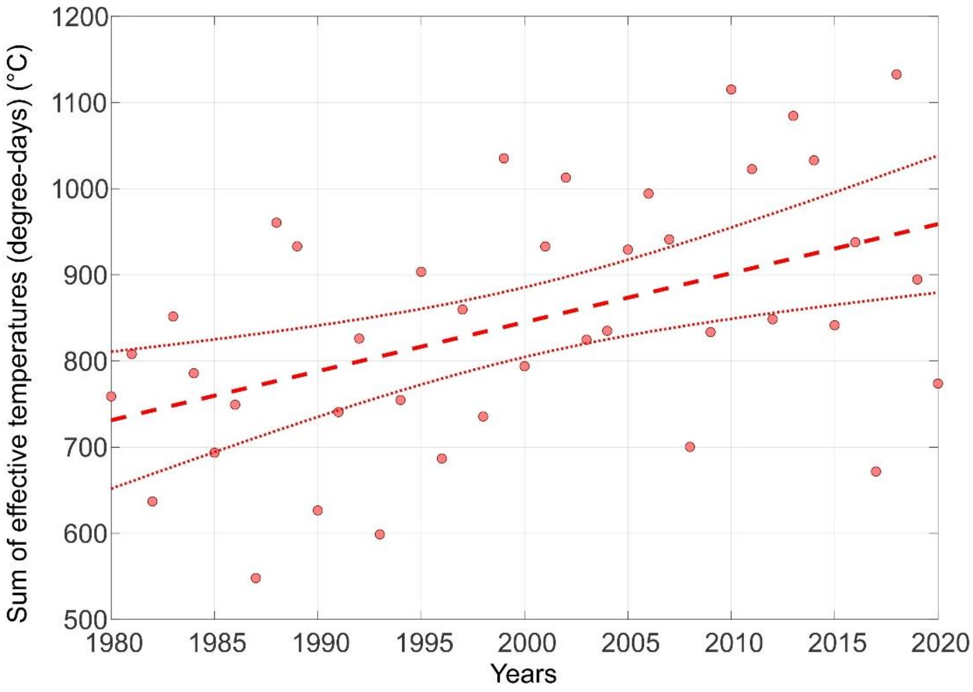

Figure S2. Dynamics of the sum of effective temperatures in Saint Petersburg during growing seasons (May–September) in 1980–2020 (calculated above the lower developmental threshold of 10°C; [13]). Pink circles, calculated values; the line of regression (based on the least squares; y=5.69x+726, where y – sum of effective temperatures (degree-days) above the lower developmental threshold of 10°C, x – year; P=0.00173); dotted lines, confidence interval (95% significance level). Correlation coefficient Rcoef=0.474. Data are taken from [15].
